# What smartphone apps exist to support recovery from opioid use disorder? A content analysis of publicly available opioid-related smartphone apps

**DOI:** 10.1186/s13722-025-00549-y

**Published:** 2025-03-13

**Authors:** Alivia Williamson, Behnam Heydarshahi, Diadora Finley-Abboud, Lili Massac, Lindsay Jacobson, Naicha Christophe, Judeline Joseph, Allison Futter, Susanne S. Hoeppner, Bettina B. Hoeppner

**Affiliations:** 1 Health through Flourishing, Department of Psychiatry, Massachusetts General Hospital, 125 Nashua Street, Boston, MA 02114 USA; 2 Harvard Medical School, Boston, MA USA; 3 Google, 1600 Amphitheatre Parkway, Mountain View, CA 94043 USA

**Keywords:** Opioid epidemic, Opioids, Opioid intervention, Opioid use disorder, mHealth, Smartphone apps

## Abstract

**Background:**

An estimated 84,181 people died due to opioid overdose in 2022 alone [1]. Mobile technologies may offer an additional pathway to provide support to people seeking recovery from opioid use disorder (OUD). To this end, we conducted a content analysis of opioid-related apps to determine to what extent apps exist that provide support to people seeking or in recovery from OUD. For apps specifically targeting OUD recovery, we identified the tools these apps offer to users seeking support in their recovery.

**Methods:**

Our team conducted a content analysis of publicly available opioid-related apps identified via web-scraping in the Apple and Google app stores. Using a two-step qualitative coding process, we first identified which apps were meaningfully related to OUD recovery and second identified what tools, if any, these apps provided.

**Results:**

Web-scraping identified 1,136 apps from the Apple App Store (*n* = 247) and Google Play (*n* = 889). Of those, 290 apps were specific to OUD recovery (65% of iOS apps, 35% of Android apps). Of those, 161 apps were included in our final analysis. The most common type of tools provided support for motivation (65.2%) and accountability (65.8%). Many apps (53%) also supported linkage to recovery support (e.g., meeting finder, telehealth). Surprisingly, fewer apps provided information about OUD recovery (43.5%) or tools for cravings (33.5%). 42.9% of apps had limited accessibility (e.g., paywalls, private invite).

**Conclusions:**

Our results show a substantial increase in the number of apps designed to support OUD recovery. Nevertheless, there remains a need for apps that provide empirically supported information and tools. Furthermore, restrictions in accessibility (i.e., findability, cost, private) may limit the impact of available apps.

**Supplementary Information:**

The online version contains supplementary material available at 10.1186/s13722-025-00549-y.

## Background

The opioid epidemic continues to take lives, with 84,181 people dying due to opioid overdose in 2022 alone [1]. Preliminary data from 2023 indicate that the rate for overdose deaths may be trending down (81,083 estimated opioid overdose deaths in 2023), however, the number of opioid overdose deaths still remains unacceptably high [1]. Moreover, the use of non-prescribed opioids remains high with an estimated 8.9 million people ages 12 years or older engaging in this behavior in 2022 [2]. The U.S. federal government has implemented several strategies to address the opioid epidemic, including expanding access to evidence-based treatment and harm reduction efforts, advancing racial equity in drug policy, reducing the supply of illicit substances, and expanding access to recovery support services [3]. Mobile technologies may offer an additional pathway to provide support to people seeking recovery from opioid use disorder (OUD).

Smartphone apps have wide reach. It is estimated that around 85% of American adults own a smartphone, with equitable ownership rates across racial and ethnic categories (85% for White, 83% for Black, and 85% for Hispanic individuals) [4, 5]. Among people with substance use disorder (SUD) rates of ownership are varied, but also high (between 64% and 94%) [6–8]. Given this reach, mobile health (mHealth) interventions are being developed in various healthcare contexts including, for example, to manage chronic illnesses like diabetes and obesity [9]. This demonstrates the societal acceptance of mHealth interventions as a tool to provide healthcare related support. mHealth interventions have been shown to have high usability, feasibility, and acceptability for supporting adherence to treatment for chronic diseases [9, 10]. Regarding substance use, research has documented substantial interest in mHealth technology, as evidenced by high download numbers for smoking cessation apps and apps targeting problematic drinking [11, 12]. 

Research investigating the efficacy of these apps to achieve their intended goals is still in progress [13–18]. To date, only three substance use apps have demonstrated their effectiveness in large-scale efficacy trials: for smoking cessation [11], alcohol recovery [19], and as adjunctive treatment for OUD [20, 21]. Another randomized controlled trial is currently underway for the app “OptiMAT,” an adjunctive intervention which provides users with tools to self-monitor daily opioid use, opioid craving, and mood [22]. Together, these findings suggest that mHealth apps hold great promise as accessible, efficacious tools to support people in overcoming problematic substance use.

To address problematic opioid use specifically, several smartphone apps are currently under development. Of these, four apps have been examined empirically to date. The “Boulder Care” app was examined to evaluate the usability of its current and proposed features [13]. Participants rated the six major features (e.g., in-app appointments, video and chat features) as helpful (median score 5/5) and rated the five proposed features (e.g., medication reminders, medication schedule) as useful (median score 5/5) [13]. Next, the “HOPE” app provides a variety of features including daily prompts for monitoring mood, stress, treatment adherence, and substance use; tracking goals and triggers; and secure messaging to providers [18]. This app showed promising 6-month outcomes in terms of early uptake and associated retention in care for most participants (56%) [14, 18]. The app “ProCare Recovery” provides an automated contingency management approach for managing OUD [16]. To test the acceptability and usability of the app, in-depth qualitative interviews with *n* = 15 participants (*n* = 5 prescribers, *n* = 10 patients) were conducted [16]. High levels of perceived usability were demonstrated [16]. Lastly, the app, “Marigold Health,” facilitates peer support for patients using medications for opioid use disorder (MOUD) through a text-based group chat application [17]. In this pilot mixed-methods study, participants (*n* = 49) used the app for 6-weeks. Following the 6-week intervention period, 20 participants were asked to complete one-on-one interviews [17]. Results indicated that delivering peer-support through the app is feasible, acceptable, and well-received, as evidenced by high app usage (64% of participants used the app on a daily basis) and participant feedback [17]. Together, these studies demonstrate the feasibility and acceptability of leveraging smartphone apps to support people in their recovery from OUD.

People looking for recovery support via smartphone apps, however, face a staggering number of substance-related apps of varying levels of quality and utility. Five systematic content analyses have sought to describe such apps [23–27]. Of these, only two content analyses focused on apps specific to OUD. The other content analyses focused on apps for pain management [27], apps for opioid dosage calculations [24], and one analysis systematically evaluated the functionality, aesthetics, and quality of information of apps that target substance use in general, and included only six OUD apps (out of 74 evaluated) [25]. This study found that all apps claimed to reduce use or promote abstinence, but had on average low overall app quality ratings (Mobile App Rating Scale (MARS) scores (M(SD) = 2.82(0.55)) [25]. 

The two remaining content analyses examined smartphone apps exclusively targeting OUD [23, 26]. Nuamah et al. (2020) searched both the Apple App Store and Google Play in May 2019 and identified 72 OUD-relevant apps (*n* = 13 Apple only; *n* = 17 Android only; *n* = 42 Both). They found that clinician-facing apps were the most common (*n* = 31, 43%), followed by apps that could be used by a general audience (i.e., patients, caregivers, or unspecified audiences; *n* = 23, 32%) [23]. Apps targeting patients were the least common (*n* = 25, 25%) [23]. Vilardaga et al. (2020) looked at apps available on Google Play in April 2020. They identified 59 apps, the majority of which were patient-facing (*n* = 36, 61%), and targeted treatments for OUD (49%) [26]. Vilardaga et al. reviewed apps only available in Google Play which limited their search compared to Nuamah et al. and yet they still documented a 40% increase in patient facing apps in just one year. This difference in “patient-facing” apps available to the public in such a brief period depicts the fast-growing nature mHealth interventions. Given the expansion of mHealth technology since the pandemic (i.e., the proliferation of telehealth), there is a need to update our knowledge on the quantity and nature of opioid-related apps that currently exist in the public domain. Moreover, as people increasingly seek health-related support via smartphone technology, including support to overcome problematic substance use, it is important to know what types of tools they encounter in the app space.

To this end, we conducted a content analysis of opioid-related apps to determine to what extent apps exist that provide support to people seeking or in recovery from OUD. Our goal was to describe the types of tools these apps offer to support people in navigating recovery from OUD. Understanding what types of tools exist in the public domain via accessible technology can help clinicians connect their patients with tools they may find helpful. Additionally, identifying gaps and limitations in currently available tools can guide future efforts to more effectively leverage smartphone app technology to provide support to people navigating recovery from OUD.

## Methods

### Sample

Smartphone apps targeting OUD were identified via web-scraping using the same keywords used in the most recent content analysis of opioid-related smartphone apps [26], namely: *opioid use disorder*,* opioid abuse*,* opioid misuse*,* opioid addiction*,* prescription opioid misuse*,* prescription opioid abuse*,* opioid abuse treatment*,* opioid abuse intervention*,* opioid abuse therapy*,* opioid abuse management*,* and opioid addiction recovery*. Web scraping is the process of using a program to extract data from websites. Oftentimes, websites provide an Application Programming Interface (API) for extracting data. This is not the case for Google Play or the Apple App Store. Thus, a member of our team (BHe) developed a web application based on two third-party Node.js libraries [Google-play-scraper] and [App-store-scraper] to perform the web scraping. This web application is called “AppSearch” and is publicly available at https://still-dusk-89361.herokuapp.com.

As user-specific profiles can influence the results displayed by Google Play and the Apple App Store, six team members independently conducted the web-scraping. Each team member conducted a search using the specified search terms (i.e., eleven searches per team member), and the results were combined. Members conducted the web-scraping between 6/3/2022-6/9/2022 for Apple apps, and 7/29/2022-8/5/2022 for apps available on Android platforms. We limited each search to two hundred results because in using search engines, such as those used to navigate app stores, most people rarely go beyond the first page of results, which typically presents approximately 10 search results [28]. Thus, including the first 200 search results is far more inclusive than typical search behaviors. Moreover, when inspecting our search results, not all search terms generated 200 results. For those which did, search results became less relevant as you moved farther from the first search result.

For each identified app, the following information was extracted via web-scraping: title of the app; name of the app developer; app ID, which is a unique identifier for mobile apps; the short summary text about the app displayed in the app store; the detailed summary text about the app displayed in the app store; the average rating the app received on the app store; the number of ratings that contributed to this score; the file size of the app (only reported in Apple App Store results); the estimated minimum number of installs to date for the app (only reported in Google Play results); date of the most recent update of the app; content rating of the app (for Apple App Store results, categories were: 4+, 9+, 12+, and 17+; for Google Play results, categories were: Everyone, Everyone 10+, Teen, Mature, Adults-Only); the price to download the app (in US dollars; this number did not include any in-app costs); and the app’s URL.

### Content analysis coding, step 1: identifying OUD recovery apps

An initial review of the web scraping results indicated that not all identified apps were meaningfully related to OUD recovery. Thus, we used a qualitative coding process to identify which apps were meaningfully related to OUD recovery, and which ones were not. To this end, we created a codebook consisting of seven categories (Supplementary Material 1), which we iteratively updated: 1: OUD Recovery– an app that provides support or guidance for the app user, who is navigating the process of recovery from OUD; 2: Caregiver– an app that provides support or guidance for the app user, who is a caregiver of a person in OUD recovery; 3: Overdose - an app that provides information or guidance for the app user on opioid overdoses, and what to do when encountering a person experiencing an opioid overdose; 4: Dosage - an app that provides support or guidance for the app user, who needs to understand correct dosing for opioids, be it for the treatment of pain or for other purposes; 5: Pain - an app that provides support or guidance for the app user, who is interested in opioids in order to leverage them for the treatment and management of pain; 6: Other– an app that does address opioids, but does not fit into any of the categories above; and 7: No– an app that the search identified, but that, upon review is determined NOT to be related to opioids in a significant way. Two independent coders reviewed each app and categorized the app as belonging to one of these seven categories, based on the information displayed about the app in the app store (i.e., description provided by the app developer and screenshots), in line with the user experience when choosing which app to download. Discrepancies between raters were resolved by consensus rating in a larger group.

### Content analysis coding, step 2: identifying OUD recovery tools provided by OUD recovery apps

In going through the process of identifying OUD recovery apps in Step 1, team members were exposed to the various tools these apps provided. From this experience, we created a codebook to describe the tools offered by each app. Here, we distinguished between tools that served to provide information; linkage to recovery support services; motivational content and tools; accountability; and tools for navigating and tracking cravings. We also noted if apps had any access restrictions. As in Step 1, two independent coders reviewed each OUD recovery app and answered yes or no questions about the contents of the app (Supplementary Material 2), using the same materials as before (i.e., information displayed about the app in the app store). Discrepancies between raters were resolved by consensus rating in a larger group, and the codebook was updated as needed. The final codebook consisted of thirty-one yes or no items.

### Analytic strategy

To describe the apps in terms of their web-scraped information (i.e., size, price, content rating, recency of updates, score, number of ratings, and minimum number of installs) and content analysis coding, we used descriptive statistics (i.e., means with standard deviations or medians with interquartile ranges for continuous variables, percentages and counts for categorical variables). To test for differences between Android vs. Apple apps, we used independent t-tests for continuous variables, and chi-square tests for categorical variables. For these comparisons, we grouped apps existing in both app stores (*n* = 22) as iOS apps. We grouped apps in this manner for two reasons, first, there were too few apps to analyze them separately (i.e., statistical power would be very low for detecting group differences). Second, the process of registering an app in the Apple App store is more restrictive than for Google Play [29]. Thus, apps listed in both stores were in compliance with the higher level of standards imposed by the Apple App Store and could be logically grouped with the iOS apps.

## Results

Web-scraping identified 1,137 apps from the Apple App Store (*n* = 247) and Google Play (*n* = 890). Of these, we excluded 12 apps (1.1%) from the first round of coding because the URL did not work at the time of rating (*n* = 5), the app was not in English (*n* = 5), or the app was specific for another country other than the U.S. (*n* = 2). The remaining apps (*n* = 1,125, 98.9%) were included in first-round coding.

Of those 1,125 apps, 774 (68.8%) were found not to be related to opioids in any significant way. Note that searches in the Google Play yielded more results unrelated to opioids in any significant way than searches in the Apple App Store (84.1% vs. 14.2%, χ^2^ (1) = 439.491, *p* < 0.0001).

**Fig. 1:**
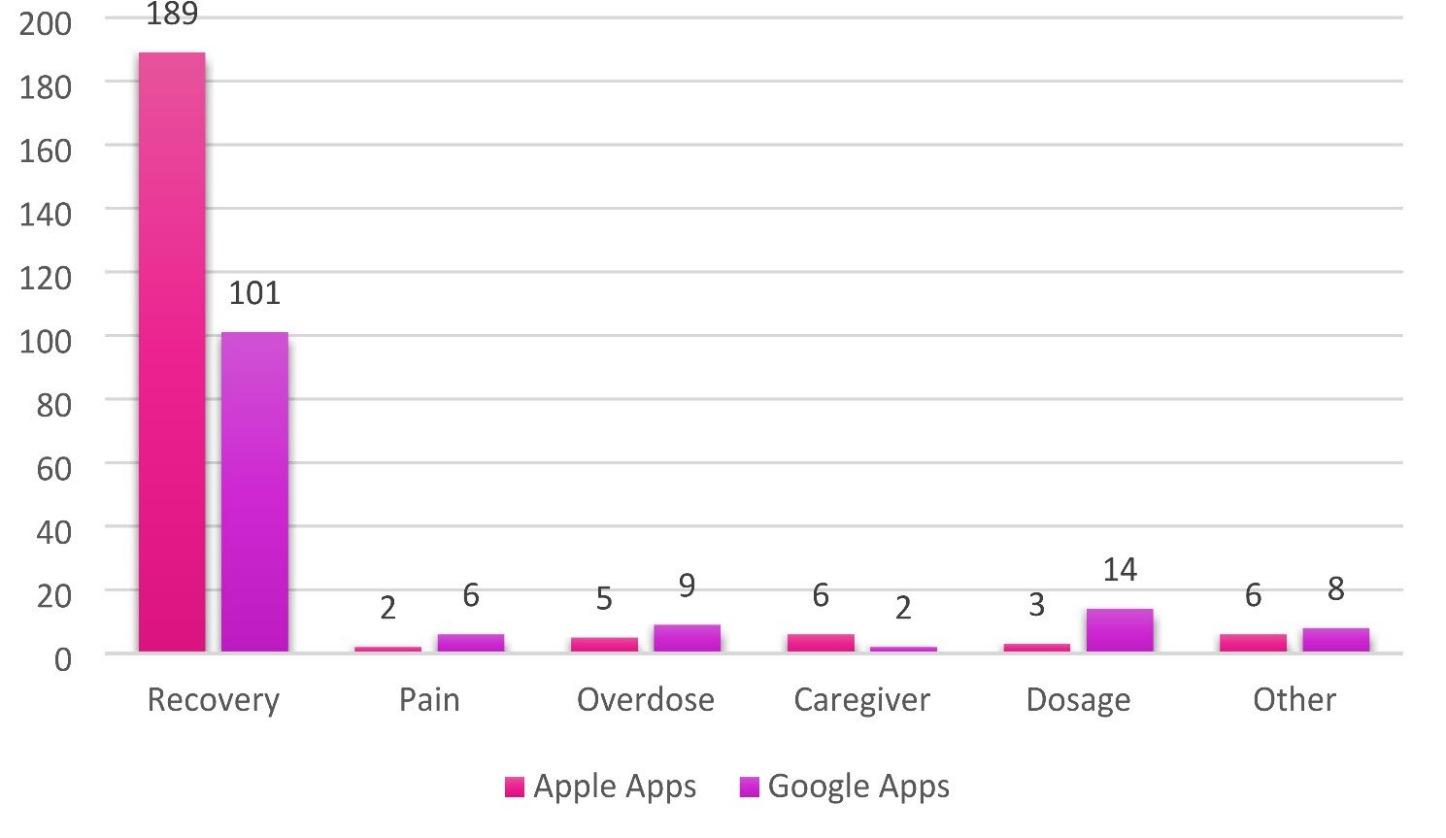
Type of opioid-related apps identified via web-scraping for Apple and Android smartphones (*n* = 351). *Note.* Total number of apps identified for each category from Apple and Android app searches

The remaining 351 apps (31.2% of total apps; Fig. 1) were related to opioids. The majority of the opioid-related apps were found to provide support or guidance for the app user, who is navigating the process of recovery from OUD (*n* = 290; 82.6%). The other apps were related to opioids but were designed for other end users: 17 apps (4.8%) provided dosage calculators or opioid conversion; 14 apps (3.99%) addressed opioid overdose; 8 apps (2.3%) addressed caregivers; 8 apps (2.3%) addressed pain management; and 14 apps (4.0%) were related to opioids but did not fit into any of these categories.

### Characteristics of apps that provide tools to support recovery from OUD

In coding the 290 apps identified in Step 1 as seeking to help the app user navigate the process of recovery from OUD, we encountered several duplicate apps (*n* = 78), that is, apps that had different names, but identical descriptions and screenshots. For one app, there were fifty-three variations of the same app, where each variation had a different name to reflect a different treatment center that offered this app via their subscription to the app provider. Other apps had different names in the Apple App Store and Google Play (*n* = 15) or were listed multiple times in the same app store with different names (*n* = 10). For our summary statistics describing the contents of the apps, we excluded duplicates from analyses. We also excluded apps from analyses if they were no longer available in the app stores when our team tried to code them (*n* = 35), if they turned out not to be related to OUD recovery (*n* = 12), or if they were related to OUD recovery but did not provide any tools (*n* = 4). For example, one app mentioned it could help users with a variety of mental health issues and addictions but did not provide any information about what tools or content the app provided.

Our search resulted in 161 unique apps that provided tools to support recovery from OUD (Table 1). Most apps (94.4%) were freely available, with no cost to download the app. For those apps that did have a cost to download, the average cost was $6.66 (SD=$3.71). Apple apps were on average more expensive than Android apps (M=$7.13, SD=$4.10 and M=$4.99, SD=$1.41, respectively). We did not conduct a group comparison regarding price as very few app (*n* = 9) had costs associated with downloading. Many apps evidenced current maintenance: 46.0% (*n* = 74) of apps had been updated within the past 6 months. A substantial number of apps, however, lacked recent updates: 25.5% (*n* = 41) had not been updated in two years.

**Table 1:** App characteristics and user appraisals of apps providing OUD recovery support (*n* = 161)

	Total n = 161		iOS n = 104		Android n = 57		Group Difference
	M / % / Md	(SD/n/IQR)	M / % / Md	(SD/n/IQR)	M / % / Md	(SD/n/IQR)	p
App Characteristics							
Size, MB, M(SD) ^a^	-	-	65.1	(48.84)	-	-	-
Price ^b^							
apps with no cost, %(n)	94.4	(152)	93.3	(97)	96.49	(55)	0.49
if any cost, price in $, M(SD)	$6.66	$3.71	$7.13	$4.10	$4.99	$1.41	
Content Rating 17+/Mature 17+, %(n)	29.8	(48)	41.3	(43)	8.8	(5)	<.0001
Recency of updates, %(n)							
updated within the past 6 months	46.0	(74)	50.0	(55)	38.60	(22)	0.19
updated more than 2 years ago	25.5	(41)	20.2	(21)	35.09	(20)	0.06
App user appraisals							
App rating score, range 1(worst) to 5 (best), M(SD)	3.56	(1.9)	3.80	(1.8)	3.11	(2.0)	0.03
Number of reviews, Md [IQR]	21.0	[0, 326]	11.5	[0, 184]	75.0	[0, 611]	0.38
Min number of installs, Md [IQR] ^c^	-	-	-	-	5000.0	[1,000, 50,000]	-

Note: *n* = 15 apps had versions available on Android and Apple platforms; when both existed, the Apple values were used. For group comparisons, apps that had versions available on Android and Apple platforms were counted as Apple apps; ^a^ = not reported for Android apps; ^b^ = Price indicates initial cost to download app, does not include payment for in-app content; ^c^ = not reported by the Apple App Store; Google Play only reported minimum installs.

Content ratings differed between apps from the Apple App Store (i.e., iOS apps) and from Google Play (i.e., Android apps). Few of the Google Play apps had content restrictions limiting them to only be available to those 17 years or older, while many of the Apple apps were designated in this way (8.8% vs. 41.3%, χ^2^ (1) = 18.48, *p* < 0.0001).

On average, apps from both app stores received high user ratings, with an average rating (M(SD)) of 3.6(1.9) stars out of five. Notably, these ratings were based on a median of 21 reviews per app. Apple apps were rated more favorably (3.8(1.8) vs. 3.1(2.0) stars; t = 0.89, *p* = 0.026).

Download numbers were only available for Android apps. The median number of minimum installs across these apps was 5,000, with a range of 1,000 to 50,000.

### Tools provided in the apps

The most common type of tools provided in the apps were tools to support motivation (*n* = 105, 65.3%) and accountability (*n* = 106, 65.8%; Table 2). To support motivation, apps provided motivational messages (*n* = 83, 51.6%; e.g., supportive quotes, bible verses, Narcotics Anonymous testimonials) and acknowledgements for staying on track with recovery (*n* = 56, 34.8%). Fewer apps asked app users to set goals related to their OUD recovery (*n* = 23, 14.3%; e.g., attend mutual help meetings, connect with sponsor, apply for employment). To support accountability, apps often provided tools to track and calculate recovery related information (*n* = 75, 46.6%; e.g., days sober, money saved).

**Table 2:** Tools provided by apps to support people in recovery from OUD (*n* = 161)

			Total		iOS		Android		Group
			*n* = 161		*n* = 104		*n* = 57		Difference
			%	(n)	%	(n)	%	(n)	p
*Information provision (% Yes)*			43.5	(70)	48.1	(50)	35.1	(20)	0.14
	Provides any information (even light info) about management and/or treatment for opioid use disorder (OUD)		34.8	(56)	39.4	(41)	26.3	(15)	0.12
	Provides information about medications for opioid use disorder (MOUD)		7.5	(12)	9.6	(10)	3.5	(2)	0.22
	Provides information about addiction		13.0	(21)	10.6	(11)	17.5	(10)	0.23
	Provides advice on how to deal with relapse		3.1	(5)	2.9	(3)	3.5	(2)	1.00
*Linkage (% Yes)*			53.4	(86)	60.6	(63)	40.4	(23)	0.02
	Connects the app user with community resources to support recovery		8.1	(13)	10.6	(11)	3.5	(2)	0.14
	Facilitates telehealth meetings regarding OUD		14.9	(24)	18.3	(19)	8.8	(5)	0.16
	Facilitates peer recovery online meetings		13.7	(22)	18.3	(19)	5.3	(3)	0.03
	Identifies nearby meetings for mutual help groups		16.1	(26)	18.3	(19)	12.3	(7)	0.38
	Connects the user with a pre-existing online community outside of the		6.2	(10)	3.8	(4)	10.5	(6)	0.17
	The app itself provides/builds an online community for peer support		26.7	(43)	33.7	(35)	14.0	(8)	0.01
*Motivational Content and Tools (% Yes)*			65.2	(105)	66.3	(69)	63.2	(36)	0.73
	Provides motivational messaging		51.6	(83)	48.1	(50)	57.9	(33)	0.25
	Provides encouragement / rewards for staying on track with recovery		34.8	(56)	36.5	(38)	31.6	(18)	0.61
	Engages app users about personal reasons to quit substance use		8.1	(13)	8.7	(9)	7.0	(4)	1.00
	Asks the app users to set goals related to their OUD recovery		14.3	(23)	16.3	(17)	10.5	(6)	0.36
*Accountability (% Yes)*			65.8	(106)	69.2	(72)	59.6	(34)	0.23
	Serves as a tool to stay accountable while navigating recovery		26.1	(42)	28.8	(30)	21.1	(12)	0.35
	Provides reminders not to use		1.9	(3)	1.9	(2)	1.8	(1)	1.00
	Provides reminders to use recovery support tools available in the app		11.8	(19)	13.5	(14)	8.8	(5)	0.45
	Checks in with the app user regarding their recovery		17.4	(28)	21.2	(22)	10.5	(6)	0.13
	Tracks and calculates recovery related information		46.6	(75)	47.1	(49)	45.6	(26)	0.87
	Functions as a journal for recovery-related journaling		18.6	(30)	19.2	(20)	17.5	(10)	0.84
*Tools for Tracking and Navigating Cravings (% Yes)*			34.8	(56)	39.4	(41)	26.3	(15)	0.12
	Provides a tracker for cravings		6.8	(11)	7.7	(8)	5.3	(3)	0.75
	Helps identify places to avoid		1.9	(3)	2.9	(3)	0.0	(0)	0.55
	Notifies app user when they are near self-identified areas to avoid		0.6	(1)	1.0	(1)	0.0	(0)	1.00
	Offers distraction tools		6.8	(11)	7.7	(8)	5.3	(3)	0.75
	Nudges app user to engage in substance-free enjoyable activities, so as to stay in recovery		4.3	(7)	5.8	(6)	1.8	(1)	0.42
	Assigns/provides mindfulness exercises		11.8	(19)	13.5	(14)	8.8	(5)	0.45
	Connects the app user with emergency contacts		13.0	(21)	15.4	(16)	8.8	(5)	0.33
	Engages app users in positive psychology exercises (other than gratitude)		3.1	(5)	4.8	(5)	0.0	(0)	0.16
	Engages app user in gratitude journaling		6.2	(10)	5.8	(6)	7.0	(4)	0.74
*Limited Access (% Yes)*			42.9	(69)	51.9	(54)	26.3	(15)	0.00
	Payment necessary for any content		36.0	(58)	42.3	(44)	24.6	(14)	0.03
	App is limited to private invite		7.5	(12)	10.6	(11)	1.8	(1)	0.06

Note: *n* = 15 apps had both Android and iOS versions; when both existed, app was counted as app.

Apps were also frequently used to provide linkage to recovery support services (*n* = 86, 53.4%). The most common type of linkage was connecting the user to an online community that provides peer support (*n* = 43, 26.7%). Linkage to other recovery support services were also provided in apps, such as to nearby mutual help groups (*n* = 26, 16.1%) or community resources (*n* = 13, 8.1%). Apps were also used to facilitate telehealth meetings and online peer recovery meetings (*n* = 24, 14.9%). Leveraging apps to provide online peer support engagement was more common among Apple than Android apps (33.7% vs. 14.0%, χ^2^ (1) = 7.25, *p* = 0.007).

Surprisingly, fewer apps provided information about OUD recovery (*n* = 70, 43.5%). If information was provided in an app, it tended to focus on the management of and treatment for OUD. Very rarely was information provided about MOUD, advice on how to respond to relapse, or even basic information about addiction (i.e., symptoms, risk factors).

Relatively few apps provided tools for tracking or responding to cravings (*n* = 56, 34.8%). The tools offered here included setting up emergency contacts, engaging the app user in mindfulness exercises, or offering distractions (Table 2). Helping app users keep track of their triggers was rare (*n* = 11, 6.8%).

### Limited access

While most apps were free to download, many apps (*n* = 58, 36%) required payment for accessing content in the app once downloaded. This was more common among Apple than Android apps (42.3% vs. 24.6%, χ^2^ (1) = 4.97, *p* = 0.026). Further limiting the reach of these apps, some apps could only be downloaded with a private invitation usually from the treatment center or recovery organization associated with the app (*n* = 12, 7.8%).

## Discussion

This content analysis of opioid-related apps available to the public via the Apple App Store and Google Play provides insight into the experience people may have as they search for app support to help them navigate their recovery from OUD. Notably, our content analysis shows that a large number of such apps exist. We identified 161 unique apps (in June-August 2022) that were explicitly designed to support people in their recovery from OUD. This is a substantial increase since the most recent prior content analysis, which only identified 36 patient-facing apps (on Google Play), using the same search terms [26]. This growth in availability of patient-facing OUD apps is in line with other relevant trends, including greater smartphone ownership rates [30], increasing investment in funding for research to create patient-facing apps [31], and increasing acceptability of mHealth supports for a variety of diseases [9, 10]. The proliferation of patient-facing apps supporting people in navigating recovery is also in line with the substantial shift towards using mobile technologies in the healthcare sector in the wake of the COVID-19 pandemic, which occurred both in healthcare in general [32], and in SUD care specifically [33]. 

Our data further suggest substantial interest in apps that help support people seeking or in recovery from OUD. Each of the apps we identified were downloaded 5,000 times (on median); this is a conservative estimate, using the estimated minimum number of total downloads Google Play provides. If similar download rates occur for iOS apps (these numbers are not reported by Apple), the apps we identified potentially reached an estimated 805,000 people (161 apps * 5,000 downloads each). For clinicians, this means that their patients may be among the people who are interested in trying out a smartphone app to help them with aspects of their OUD recovery. We hope that this content analysis provides insight to clinicians into the kinds of tools their patients may be able to find in the app space, so that they may be better equipped to engage with their patients in conversations about smartphone app support.

Unfortunately, our content analyses also made clear that it may be difficult for users to find “patient-facing” apps (i.e., apps for users who want to use and apply OUD recovery tools). This may be particularly true for people with Android phones, where most search results were not meaningfully related to opioids (84.1%), and even fewer to recovery from OUD. For iPhones, the same search terms resulted in more relevant apps (76.8%). This finding is troubling, because Android ownership is higher than iPhone ownership among Black individuals [34], who are disproportionately impacted by the opioid overdose crisis [35, 36]. On the other hand, the Android apps were less likely to be behind paywalls, which were more common in Apple apps.

In looking at the tools currently available, we noted several missed opportunities that appear worthwhile to consider for future app development. Most apps provided motivational and accountability support, which is reminiscent of the important ingredients underlying mutual help groups. Notably missing, however, are apps that provide OUD recovery tools used by apps shown to be efficacious [11, 19–21]. Most relevantly, the “ACHESS” app offers a “High Risk Locator,” which uses GPS location to prompt users to take action to engage in recovery support if they are near a place that could cause them to relapse [37]. In contrast, only 0.6% (*n* = 1) of apps found in the Apple App Store and Google Play in our web-scraping period (June-August 2022) offered this functionality. This suggests a science to implementation gap. Smartphone apps can be built much more quickly than they can be tested rigorously. Randomized-controlled trials take an average of five and a half years to conduct and publish findings [38], whereas the average time to develop a mobile app is around 6–9 months [39]. Our findings suggest that it is the rule rather than the exception to encounter untested apps (and untested app tools) in the public domain. It is not clear to what extent this offering of untested apps creates a potential for harm. Given the nature of these apps (e.g., motivational), it seems unlikely that using them would be harmful, except to the degree to which they prevent people from reaching out for more efficacious support. This potential could be offset by providing information within the apps about addiction and recovery support, and by providing clear information in the app’s description about the state of the science on the app. On a more positive note, there has been an increase in research into the development, acceptability, functionality, and efficacy of mHealth apps for a variety of diseases and public health issues over the last two decades [31], which means that future app developers have a growing body of research to consult when designing their apps.

Another area ripe for future development is leveraging OUD recovery apps to provide basic information about addiction and recovery (i.e., symptoms, risk, factors). Particularly lacking was information on MOUD. This is a notable absence considering MOUD are regarded as the gold standard treatment for OUD. MOUD substantially reduce the risk of overdose [40], but less than 25% of those diagnosed with OUD initiate medications [41, 42]. Moreover, early discontinuation and retention on MOUD continues to be major barriers to OUD treatment efficacy at the population level [43]. Novel approaches are needed to support persons in initiating MOUD and staying engaged in treatment. Smartphone apps present a unique avenue to provide such supports including increasing education on MOUD (i.e., types, side effects, treatment schedules), locating clinics or hospitals that provide MOUD (i.e., GPS based treatment locators), or helping those already engaged in MOUD care manage their medication adherence (i.e., medication reminders).

Promisingly, some of the identified apps included tools to deliver newly emerging therapeutic approaches. For example, several apps used mindfulness exercises. This is an approach that is used in the only smoking cessation app with demonstrated efficacy to date, demonstrating the feasibility of this approach when delivered via mHealth technologies [11]. Recently emerging research has pointed towards the promise of mindfulness approaches in recovery from OUD [44–46], underscoring the potential utility of leveraging this approach in apps to support recovery from OUD. A few apps also included tools that engaged users in gratitude journaling, a recently developed treatment approach for people in recovery from alcohol and substance use disorders [47]. 

### Limitations

There were several limitations to this content analysis. First, we used the apps’ descriptions and screenshots as shown in the app stores to code the app’s contents rather than downloading and experiencing the app. This may lead to errors if the app’s contents vary widely from the information in the app stores. Second, due to the amount of time required to code the apps, we could not include all initially identified apps in analyses because some apps became unavailable over time. This variability in the availability of apps highlights the ever-changing nature of the smartphone app offerings available to the public. Third, we were only able to extract accurate minimum estimates of the total number of downloads for Android apps, and thus could not provide actual download numbers of the identified apps. Relatedly, it is also important to note that while download numbers indicate interest in an app, the number of downloads an app generates does not necessarily equate to the number of users engaging with the app’s content long term. Research examining the rates of app abandonment find that around 71% of apps in general are abandoned in the first three months [48]. This can be due to a variety of reasons including app functionality (e.g., battery drain, incompatibility with phone, lack of desired features) and more personal reasons (e.g., no longer needing the support the app provides, or no longer attempting to achieve the initial health goal) [49]. Lastly, our team encountered multiple apps that were accessible only by invitation from a private organization or required payment once you downloaded the app (i.e., tele-health apps that required users to use insurance or apps that have subscription fees). Some of these apps would make note of this in their descriptions while others did not. This means that the percent of apps with limited access (Table 2) and the calculated mean cost of apps (Table 1) may be higher than we reported in our analysis.

## Conclusion

Overall, this content analysis shows a substantial increase in the number of smartphone apps designed to support recovery from OUD since the previous two content analyses [23, 26]. Despite this proliferation, however, there remain gaps in the market for apps that provide updated and high-quality information regarding the different treatment and support options available for OUD. Furthermore, there is a striking lack of therapeutic tools provided by these apps to help support people in their recovery. There is also an accessibility barrier, considering that around 43% of apps required payment for some or all their contents or required users to receive an invitation from a private group or organization to join. This rate of encountering paywalls is similar for apps that provide support for quitting smoking [50] or problematic drinking [12], and may be related to the considerable costs that are incurred in developing an app (anywhere from $5,000 up to $133,000) [51], and maintaining it. The restriction in access due to requiring private invites, however, is not an issue that has been previously reported as an accessibility issue for addiction-related apps. Perhaps this issue has emerged because people with OUD are more likely to be engaged in treatment than people seeking to quit smoking or problematic drinking [52, 53]. It is an unfortunate trend, however, given that a key strength of smartphone app technology is its wide reach.

mHealth scientists interested in developing app interventions for this populations should be aware of the current layout in the app market and the barriers to use for individuals interested in this type of support. Moreover, as more apps are empirically tested [11, 13–19, 22] future app developers should focus on providing evidence-based support and tools. Smartphone apps are a fast growing and novel way to provide access to treatment and support for OUD, but these apps are only helpful if they are findable, accessible, and effective. While these apps are not perfect, healthcare professionals should still consider telling their patients about the availability of such apps that could provide their patients with additional tools to support them in their recovery journey. Smartphone apps may be able to overcome barriers to traditional in-person recovery support. To help patients navigate the various apps out there, which range in quality, there are mHealth app indexes that review mHealth apps for the quality of the information and tools they provide. MIND [54] uses 105 objective questions based on the American Psychiatric Association’s App Evaluation Model to review apps. Once an app is reviewed it is added to the database and users can use the search and filter functions to find apps that fit their specific needs.

## Electronic supplementary material

Below is the link to the electronic supplementary material.

Supplementary Material 1

Supplementary Material 2

## Data Availability

All data generated or analyzed during this study are included in this published article and its supplementary files.

## Abbreviations

OUD: Opioid use disorder
SUD: Substance Use Disorder
MARS: Mobile App Rating Scale
M: Mean
SD: Standard Deviation
API: Application Programming Interface
MOUD: Medications for opioid use disorder
%: Percentage
Md: Median
N: Sample size
IQR: Interquartile range

## References

[1] National Center for Health Statistics. U.S. Overdose Deaths Decrease in 2023, First Time Since 2018: Centers for Disease Control and Prevention; 2024 [Available from: https://www.cdc.gov/nchs/pressroom/nchs_press_releases/2024/20240515.htm

[2] Delphin-Rittmon ME, The National Survey on Drug Use and Health.: 2020 2022 [Available from: https://www.samhsa.gov/data/sites/default/files/reports/slides-2020-nsduh/2020NSDUHNationalSlides072522.pdf

[3] Office of National Drug Control Policy. National Drug Control Strategy: The White House; 2022 [Available from: https://www.whitehouse.gov/wp-content/uploads/2022/09/2022-National-Interdiction-Command-and-Control-Plan-NICCP.pdf

[4] Dallery J, Defulio A, Raiff BR. Digital contingency management in the treatment of substance use disorders. Policy Insights Behav Brain Sci. 2023;10:51–8.

[5] Pew Research Center. Mobile Fact Sheet 2021 [Available from: https://www.pewresearch.org/internet/fact-sheet/mobile/

[6] Hsu M, Martin B, Ahmed S, Torous J, Suzuki J, Smartphone, Ownership. Smartphone utilization, and interest in using mental health apps to address substance use disorders: literature review and Cross-sectional survey study across two sites. JMIR Form Res. 2022;6(7):e38684. 10.2196/38684 PMC9305402 35797102

[7] Ashford RD, Lynch K, Curtis B. Technology and social media use among patients enrolled in outpatient addiction treatment programs: Cross-Sectional survey study. J Med Internet Res. 2018;20(3):e84. 29510968 10.2196/jmir.9172 PMC5861298

[8] Masson CL, Chen IQ, Levine JA, Shopshire MS, Sorensen JL. Health-related internet use among opioid treatment patients. Addict Behav Rep. 2019. 10.1016/j.abrep.2018.100157 PMC6542730 31193741

[9] Wang Y, Min J, Khuri J, Xue H, Xie B, L AK et al. Effectiveness of mobile health interventions on diabetes and obesity treatment and management: systematic review of systematic reviews. JMIR Mhealth Uhealth. 2020;8(4). 10.2196/15400 PMC7218595 32343253

[10] Hamine S, Gerth-Guyette E, Faulx D, Green BB, Ginsburg AS. Impact of mHealth chronic disease management on treatment adherence and patient outcomes: a systematic review. J Med Internet Res. 2015;17(2). 10.2196/jmir.3951 PMC4376208 25803266

[11] Bricker JB, Watson NL, Mull KE, Sullivan BM, Heffner JL. Efficacy of smartphone applications for smoking cessation: A randomized clinical trial. JAMA Intern Med. 2020;180(11):1472–80. 32955554 10.1001/jamainternmed.2020.4055 PMC7506605

[12] Hoeppner BB, Schick MR, Kelly LM, Hoeppner SS, Bergman B, Kelly JF. There is an app for that - Or is there? A content analysis of publicly available smartphone apps for managing alcohol use. J Subst Abuse Treat. 2017;82:67–73. 29021117 10.1016/j.jsat.2017.09.006

[13] Bosse JD, Hoffman K, Wiest K, Todd Korthuis P, Petluri R, Pertl K, et al. Patient evaluation of a smartphone application for telehealth care of opioid use disorder. Addict Sci Clin Pract. 2022;17:50. 36085078 10.1186/s13722-022-00331-4 PMC9462609

[14] Hodges J, Waselewski M, Harrington W, Franklin T, Schorling K, Huynh J et al. Six-month outcomes of the HOPE smartphone application designed to support treatment with medications for opioid use disorder and piloted during an early statewide COVID-19 lockdown. Addict Sci Clin Pract. 2022;17. 10.1186/s13722-022-00296-4 PMC8899792 35255965

[15] Magee MR, McNeilage AG, Avery N, Glare P, Ashton-James CE. mHealth Interventions to Support Prescription Opioid Tapering in Patients With Chronic Pain: Qualitative Study of Patients’ Perspectives. JMIR Form Res. 2021;5(5). 10.2196/25969 PMC8170552 34003133

[16] Proctor SL, Rigg KK, Tien AY. Acceptability and usability of a Reward-Based mobile app for opioid treatment settings: mixed methods pilot study. JMIR Form Res. 2022;6(10):e37474. 36197705 10.2196/37474 PMC9582914

[17] Scherzer CR, Ranney ML, Patena J, Jennings E, Langdon KJ, Beaudoin FL. Peer support for opioid use disorder: feasibility and acceptability of a moderate Text-Based group chat application. J Addict Res Ther. 2021;12(3):421.

[18] Waselewski ME, Flickinger TE, Canan C, Harrington W, Franklin T, Otero KN, et al. A mobile health app to support patients receiving Medication-Assisted treatment for opioid use disorder: development and feasibility study. JMIR Form Res. 2021;5(2):e24561. 33620324 10.2196/24561 PMC7943342

[19] Gustafson DH, McTavish FM, Ming-Yuan C, Atwood AK, Johnson RA, Boyle MG, et al. A smartphone application to support recovery from alcoholism: a randomized clinical trial. JAMA Psychiatry. 2014;71(5):566–72. 24671165 10.1001/jamapsychiatry.2013.4642 PMC4016167

[20] Campbell ANC, Nunes EV, Matthews AG, Stitzer M, Miele GM, Polsky D, et al. Internet-Delivered treatment for substance abuse: A multisite randomized controlled trial. Am J Psychiatry. 2014;171:683–90. 24700332 10.1176/appi.ajp.2014.13081055 PMC4079279

[21] Christensen DR, Marsch LA, Chopra MP, Landes RD, Jackson L, Mancino MJ, et al. Adding an Internet-Delivered treatment to an efficacious treatment package for opioid dependence. J Consult Clin Psychol. 2014;82:964–72. 25090043 10.1037/a0037496 PMC4244262

[22] Thompson RG, Bollinger M, Mancino MJ, Hasin D, Han X, Bush KA et al. Smartphone intervention to optimize medication-assisted treatment outcomes for opioid use disorder: study protocol for a randomized controlled trial. Trials. 2023;24. 10.1186/s13063-023-07213-3 PMC10071730 37016394

[23] Nuamah J, Mehta R, Sasangohar F. Technologies for opioid use disorder management: mobile app search and scoping review. JMIR Mhealth Uhealth. 2020;8(6). 10.2196/15752 PMC7305558 32501273

[24] Haffey F, Brady RR, Maxwell S. A comparison of the reliability of smartphone apps for opioid conversion. Drug Saf. 2013;36(2):111–7. 23322549 10.1007/s40264-013-0015-0

[25] Tofighi B, Chemi C, Ruiz-Valcarcel J, Hein P, Hu L. Smartphone apps targeting alcohol and illicit substance use: systematic search in in commercial app stores and critical content analysis. JMIR Mhealth Uhealth. 2019;7(4). 10.2196/11831 PMC6658280 31008713

[26] Vilardaga R, Fisher T, Palenski PE, Kumaresan V, Mannelli P, Sweitzer MM, et al. Review of popularity and quality standards of Opioid-Related smartphone apps. Curr Addict Rep. 2020;7(4):486–96. 33777644 10.1007/s40429-020-00344-6 PMC7993400

[27] Wallace LS, Dhingra LK. A systematic review of smartphone applications for chronic pain available for download in the united States. J Opioid Manag. 2014;10(1):63–8. 24604571 10.5055/jom.2014.0193

[28] Dean B. We analyzed 4 million Google Search Results Here’s What We Learned About Organic Click Through Rate backlinko.com: BACKLINKO; 2024 [Available from: https://backlinko.com/google-ctr-stats

[29] Bigabid. Key 2023 Apple App Store vs Google Play Store Differences for Developers and Marketers Bigabid.com: Bigabid; 2023 [Available from: https://www.bigabid.com/apple-app-store-vs-google-play-store-differences/#:~:text=The%20biggest%20advantage%20of%20the,starting%20with%20the%20publishing%20process

[30] Pew Research Center. Mobile Fact Sheet pewresearch.org: Pew Research Center. 2024 [Available from: https://www.pewresearch.org/internet/fact-sheet/mobile/

[31] Hansen WB, Scheier LM. Specialized smartphone intervention apps: review of 2014 to 2018 NIH funded grants. JMIR Mhealth Uhealth. 2019;7(7):e14655. 31359866 10.2196/14655 PMC6690163

[32] Shaver J. The state of telehealth before and after the COVID-19 pandemic. Prim Care. 2022;49(4):517–30. 36357058 10.1016/j.pop.2022.04.002 PMC9035352

[33] Molfenter T, Roget N, Chaple M, Behlman S, Cody O, Hartzler B, et al. Use of telehealth in substance use disorder services during and after COVID-19: online survey study. JMIR Mental Health. 2021;8(2):e25835. 33481760 10.2196/25835 PMC7895293

[34] Desilver D. As it turns 6, a look at who uses the iPhone (no, not everybody) Pewresearch.org: Pew Research Center; 2013 [Available from: https://www.pewresearch.org/short-reads/2013/06/29/as-it-turns-6-a-look-at-who-uses-the-iphone-no-not-everybody/

[35] Lippold KM, Jones CM, Olsen EOM, Giroir BP. Racial/ethnic and age group differences in opioid and synthetic opioid–involved overdose deaths among adults aged ≥ 18 years in metropolitan areas—United States, 2015–2017. MMWR Morb Mortal Wkly Rep. 2019;68:967–73. 31671083 10.15585/mmwr.mm6843a3 PMC6822810

[36] Friedman JR, Hansen H. Evaluation of increases in drug overdose mortality rates in the US by race and ethnicity before and during the COVID-19 pandemic. JAMA Psychiatry. 2022;79:379–81. 35234815 10.1001/jamapsychiatry.2022.0004 PMC8892360

[37] Ford IIJH, AE, Dinauer S, Johnson KA, Pe-Romashko K, Gustafson DH. Successful organizational strategies to sustain use of A-CHESS: A mobile intervention for individuals with alcohol use disorders. J Med Internet Res. 2015;17(8). 10.2196/jmir.3965 PMC4642385 26286257

[38] Pham Q, Wiljer D, Cafazzo JA. Beyond the randomized controlled trial: A review of alternatives in mHealth clinical trial methods. JMIR Mhealth Uhealth. 2016;4(3):e107. 27613084 10.2196/mhealth.5720 PMC5035379

[39] Yaroslav Titenok. How Long Does it Take to Build an App in 2024: Development Time and Expert Tips sloboda-studio.com: Sloboda Studio; 2024 [Available from: https://sloboda-studio.com/blog/how-long-does-it-take-to-make-an-app/#:~:text=Average%20mobile%20app%20development%20time%20can%20take%206%20to%209%20months.%26;text=However%2C%20as%20practice%20shows%2C%20each,average%20at%20various%20project%20stages

[40] Volkow ND, Collins FS. The role of science in addressing the opioid crisis. N Engl J Med. 2017;377(4):391–4. 28564549 10.1056/NEJMsr1706626

[41] Morgan JR, Schackman BR, Leff JA, Linas BP, Walley AY. Injectable Naltrexone, oral Naltrexone, and buprenorphine utilization and discontinuation among individuals treated for opioid use disorder in a united States commercially insured population. J Subst Abuse Treat. 2018;85:90–6. 28733097 10.1016/j.jsat.2017.07.001 PMC5750108

[42] Hadland SE, Wharam FJ, Schuster MA, Zhang F, Samet JH, Larochelle MR. Trends in receipt of buprenorphine and Naltrexone for opioid use disorder among adolescents and young adults, 2001–2014. JAMA Pediatr. 2017;171(8):747–55. 28628701 10.1001/jamapediatrics.2017.0745 PMC5649381

[43] Morgan JR, Walley AY, Murphy SM, Chatterjee A, Hadland SE, Barocas J et al. Characterizing initiation, use, and discontinuation of extended-release buprenorphine in a nationally representative united States commercially insured cohort. Drug Alcohol Depend. 2021:108764. 10.1016/j.drugalcdep.2021.108764 PMC8488795 34051547

[44] Garland E, Hanley AW, Kline A, Cooperman NA. Mindfulness-Oriented recovery enhancement reduces opioid craving among individuals with opioid use disorder and chronic pain in medication assisted treatment: ecological momentary assessments from a stage 1 randomized controlled trial. Drug Alcohol Depend. 2019;203:61–5. 31404850 10.1016/j.drugalcdep.2019.07.007 PMC6939880

[45] Cooperman NA, Hanley AW, Kline A, Garland EL. A pilot randomized clinical trial of mindfulness-oriented recovery enhancement as an adjunct to methadone treatment for people with opioid use disorder and chronic pain: impact on illicit drug use, health, and well-being. J Subst Use Addict Treat. 2021;127. 10.1016/j.jsat.2021.108468 PMC8281569 34134880

[46] Zullig KJ, Lander LR, Sloan S, Brumage MR, Hobbs GR, Faulkenberry L. Mindfulness-based relapse prevention with individuals receiving medication-assisted outpatient treatment for opioid use disorder. Mindfulness. 2018;9(2):423–9.

[47] Krentzman AR, Hoeppner BB, Hoeppner SS, Barnett NP. Development, feasibility, acceptability, and impact of positive psychology journaling intervention to support addiction recovery. J Posit Psychol. 2023;18(2):573–91. 10.1080/17439760.2022.2070531 PMC12346788 40809683

[48] Mondal J, Chakrabarti S. The abandonment behaviour of the branded app consumer: A study using interpretive structural modelling approach. J Retailing Consumer Serv. 2021;63.

[49] Murnane EL, Huffaker D, Kossinets G. Mobile health apps: adoption, adherence, and abandonment. Adjunct Proceedings of the 2015 ACM International Joint Conference on Pervasive and Ubiquitous Computing and Proceedings of the 2015 ACM International Symposium on Wearable Computers; Osaka, Japan: Association for Computing Machinery; 2015. pp. 261–4.

[50] Hoeppner BB, Hoeppner SS, Seaboyer L, Schick MR, Wu GW, Bergman BG, et al. How smart are smartphone apps for smoking cessation?? A content analysis. Nicotine Tob Res. 2016;18(5):1025–31. 26045249 10.1093/ntr/ntv117 PMC5942604

[51] Artem Digtiev. App Development Cost. (2024) businessofapps.com: Business of Apps; 2024 [Available from: https://www.businessofapps.com/app-developers/research/app-development-cost/

[52] Deboarah Dowell S, Brown S, Gyawali J, Hoenig J, Ko C, Mikosz et al. Treatment for Opioid Use Disorder: Population Estimates — United States. 2024. 10.15585/mmwr.mm7325a1 PMC11254342 38935567

[53] Substance Abuse and Mental Health Services Administration. 2021 National Substance Use And Mental Health Services Survey (N-SUMHSS) Detailed Tables: Substance Abuse and Mental Health Services Administration; 2023 [Available from: https://www.samhsa.gov/data/report/2021-nsumhss-detailed-tables

[54] MIND. M-Health Index. & Navigation Database [Available from: https://mindapps.org/

