# Supplementary material for: What smartphone apps exist to support recovery from opioid use disorder? A content analysis of publicly available opioid-related smartphone apps
Source: Addict Sci Clin Pract. 2025 Mar 13;20:26. doi: 10.1186/s13722-025-00549-y (PMC11905484; doi:10.1186/s13722-025-00549-y)
Supplement: Supplementary file 1 — Supplementary Material 1 [file 13722_2025_549_MOESM1_ESM.docx]

**Codebook for the Initial Categorization of Apps**

For each app on the list, please rate what type of app this is. Keep in mind that an app is supposed to provide a tool of some sort. So, when coding an app, ask yourself: “what does this app do?” It can provide information, it can help the user locate a treatment provider, or act as an in-your-pocket coach. With this in mind, code which category of app this is. If an app is targeting multiple groups (e.g., person in recovery and caregiver), code as belonging to the category appearing first in this codebook (e.g., in this example, the code would be “recovery”).

- **Recovery** – **an app that provides support/guidance for the app user, who is navigating the process of recovery from opioid use disorder.** The description of the app must use the word addiction or synonym thereof (e.g., substance use disorder; recovery, sobriety). Include an app in this category if it provides any of the tools below. Oftentimes, alcohol use disorder is used as a guide to inform approaches for opioid use disorder. **Include an app in this category, if the tools offered could be used for recovery from any substance use, including opioid use**. **Include the app, if it targets addiction and does not exclude opioids.** **Exclude an app from this category, if it specifically targets a non-opioid substance (e.g., alcohol) to the exclusion of opioid use disorder (e.g., interface specifically asks for # of drinks, or in other ways is very specific about a non-opioid substance) or non-substance use addictions (e.g., gambling, porn).** Note that these recovery apps need not be science-based. They also do not need to apply to everyone with OUD, so long as they provide tools to support OUD recovery for some (e.g., an OUD recovery app for LGBTQ folk; a scripture-based app for Christians with OUD). Here are some examples of tools that such apps may provide to support the app user in their recovery from OUD:
  - Provides information about management and/or treatment for opioid use disorder (OUD), including information about OUD doctors, therapies, support group, etc. (e.g., teaches you about the brain in addiction and recovery).
  - Provides information about medications for opioid use disorder (MOUDs).
  - Facilitates telehealth meetings regarding OUD.
  - Involves a doctor to facilitate the connection to the app.
  - Provides motivational messaging, that is, content provided by the app to the app user (e.g., recovery quotes, inspirational quotes).
  - Connects the user with an online community (e.g., to sharing progress, peer mentor).
  - Teaches about opioid use triggers and solutions to these triggers.
  - Providers reminders not to use, and/or to use recovery support tools.
  - Connects the app user with a recovery-supportive care team.
  - Identifies nearby meetings for mutual help groups (e.g., AA, SMART, etc.).
  - Checks in with the app user regarding their recovery (e.g., questions to assess how you feel).
  - Tracks and calculates recovery related information (e.g., counting days of not using, keeping track of daily recovery tasks, money saved).
  - Helps identify places to avoid.
  - Raises awareness and nudges app use to engage in enjoyable activities, so as to stay in recovery.
  - Provides advice on how to deal with relapse.
  - Provides encouragement / rewards for staying on track with recovery (e.g., badges).
  - Serves as a tool to stay accountable while navigating recovery.
  - Serves as a “rescue” tool to help connect the app user with help when they need it regarding their OUD recovery.
  - Functions as a journal for recovery-related journaling (e.g., entering your own motivational quotes, ideas that motivate you).
  - Assigns mindfulness exercises.
  - Engages app users about personal reasons to quit substance use.
  - Offers distraction tools.
  - Provides information about addiction (e.g., symptoms, risk factors).
  - Nudges the app users to set (and strive for) goals related to their OUD recovery.
  - Provides a tracker for cravings.
  - Provides mental health support (e.g., for anxiety, depression). **Only include in this category, however, if OUD, SUD, or addiction is specifically mentioned in the app description.**
- **Caregiver** – **an app that provides support/guidance for the app user, who is a caregiver of a person in OUD recovery**. Caregiver is loosely defined, and includes anyone who considers themselves affected by the OUD of someone in their personal network (e.g., parents, siblings, children, friends of those with OUD). **Include apps in this category that target caregiving stress in a broader sense, which includes addiction (e.g., an app targeting caregiver stress for those caring for sick children or a spouse with addiction).** **Exclude an app from this category if the focus is exclusively on such other groups (e.g., just on parents of sick children) or target professional caregivers (e.g., clinicians, nurses, but include AA sponsors and peer coaches, so long as the app focuses on their personal relationship with the index person).** The index person (i.e., the person with OUD who is being supported by the app user) may or may not be in recovery (i.e., they have a problem with their opioid use, but may or may not be taking steps to address these problems). Tools that such apps may provide are the same as those provided above (i.e., recovery), but these tools are leveraged to understand and support someone else’s recovery, and to engage in self-care to be able to manage the stress of supporting someone else in recovery**.**
  - Provides information about addiction and recovery.
  - Provides a platform for interacting with other caregivers.
  - Provides the space and tools to digest & process what they’re going through with others who relate.
- **Overdose** - **an app that provides information/guidance for the app user on opioid overdoses, and what to do when encountering a person experiencing an opioid overdose**. This type of app may be designed for a variety of people, including for healthcare professionals, trainees, or the public.
  - Teaches the app user about naloxone (aka Narcan), which is used as an overdose reversal medication.
  - Provides steps of how to deal with an overdose, and what to do during an overdose event.
  - Provides medical information about what happens during an overdose event, and what medical procedures happen at this time.
  - Connects the app user with overdose response teams (i.e., connect to nearest ER or other emergency services).
  - Provides information on how to prevent an opioid overdose.
  - Provides tracking information that tracks overdoses occurring in a community.
  - Provides overdose relevant information (e.g., NARCAN supply distribution, available mental health services) to harm reduction organizations.
- **Dosage** - **an app that provides support/guidance for the app user, who needs to understand correct dosing for opioids, be it for the treatment of pain or for other purposes**. This type of app may be designed for a variety of people, including for healthcare professionals, trainees, or the public. Here are some examples of tools that such apps may provide to help the app user in calculating and/or understanding opioid dosages:
  - Teaches the app user about different kinds of opioids.
  - Provides a calculator to convert opioid dosages into different metrics.
  - Provides information about different routes of administration for opioids (e.g., IVs).
  - Provides information about adverse reactions.
  - Calculates correct dosages for different usages (e.g., rescue doses).
  - An app that provides guidance around bad batches of fentanyl (e.g., Apple app “SOAR Central Ohio”).
- **Pain** - **an app that provides support/guidance for the app user, who is interested in opioids to leverage them for the treatment and management of pain.** This type of app may be designed for a variety of people, including for healthcare professionals, trainees, or the public**. Include apps as belonging into this category, if a main theme of the app is pain (e.g., an app that addresses pain and anxiety together should be coded as belonging into this category).** Here are some examples of tools that such apps may provide:
  - Provide insight into different relevant timeframe for pain management (e.g., acute pain, short-term pain, chronic pain, poster-surgery pain).
  - Provides tools and/or information on how to assess, monitor, and mange a person’s pain.
- **Other** – **an app that does address opioids but does not fit into any of the categories above**. Present your case as to why an app goes into this category to address these questions:
- Why does this app deserve a different code?
- What makes the app stand out in its own category?
- What would this new code be?

Examples of apps belonging in this category are:

- Diagnostic app that helps users test if they have a substance use disorder.
- An app that provides information about community level approaches to OUD or SUD.
- An app that provides scheduling or other administrative assistance to professional staff at substance use related centers.
- An app that provides a game to teach people about recovery (e.g., Apple app “The Drug Recovery Game”).
- **No** – **an app that the search identified, but that upon review is determined to NOT relate to opioids in a significant way**

Examples of apps belonging in this category are:

- - An app showing videos of interviews with lots of different people, including people with substance use issues.
  - An app that helps the user deal with the urge to self-harm.
  - An app that helps the user reduce their sugar intake.
  - An app that helps people with gambling addiction.
  - An app that reminds people to take their medications (any medications) on time.
  - An app that provides information about medications in general (e.g., what authentic pills look like), but is not focusing on opioids.
  - An app that addressed overdoses for opioids but is talking about overdose issues more generally with regards to hundreds of other medications.
  - An app that does calculate dosages, but not targeted/focused on opioids.
  - An encyclopedic app that could provide info about opioids but is not focused on it.
  - An app that helps with ‘bad habits’ but does not specifically target addictions (e.g., a wellness tracker that does not talk about addiction would fit this category).
