# Supplementary material for: What smartphone apps exist to support recovery from opioid use disorder? A content analysis of publicly available opioid-related smartphone apps
Source: Addict Sci Clin Pract. 2025 Mar 13;20:26. doi: 10.1186/s13722-025-00549-y (PMC11905484; doi:10.1186/s13722-025-00549-y)
Supplement: Supplementary file 2 — Supplementary Material 2 [file 13722_2025_549_MOESM2_ESM.docx]

**Codebook for Describing the Tools provided by OUD Recovery Apps**

- Information Provision
  1. Does the app provide any information (even light info) about management and/or treatment for opioid use disorder (OUD), (including information about OUD doctors, therapies, support group, teaches you about the brain in addiction and recovery)?
  2. Does the app provide information about medications for opioid use disorder (MOUDs)?
  3. Does the app provide information about addiction (e.g., symptoms, risk factors)?
  4. Does the app provide advice on how to deal with relapse?
- Connects user with treatment/ recovery options

1. Does the app connect the app user with community resources to support recovery (e.g., recovery community centers, recovery housing)?
2. Does the app facilitate telehealth meetings regarding OUD?
3. Does the app facilitate peer recovery online meetings (e.g., Zoom meetings with recovery coach)?
4. Does the app identify nearby meetings for mutual help groups (e.g., AA, SMART, etc.)?
5. Does the app connect the user with a pre-existing online community outside of the app (e.g., to sharing progress, peer mentor); the app needs to connect to an online community (e.g., Facebook group, RCC group online), and not a particular person (e.g., sending an email to a person)?
6. Does the app itself provide/build an online community for peer support?

- Motivational Content and Tools:

1. Does the app provide motivational messaging (e.g., recovery quotes, encouraging comments from others in the shared app space, including other people’s sober stories)?
2. Does the app provide encouragement / rewards for staying on track with recovery (e.g., badges, sobriety calculator that says “great job”)?
3. Does the app engage app users about personal reasons to quit substance use (i.e., prompts them to reflect upon and specify their own reasons for seeking/being in recovery, e.g., wanting to fulfill parenting role, wanting to live)?
4. Does the app ask the app users to set goals related to their OUD recovery (e.g., setting a date for starting recovery attempt, logging the intent to visit an RCC or go to a mutual help meeting; the app asking users to describe their goals)?

- Accountability

1. Does the app serve as a tool to stay accountable while navigating recovery (i.e., the app provides tools to help the app user track how they are doing in their recovery over time, e.g., a daily log)?
2. Does the app provide reminders not to use?
3. Does the app provide reminders to use recovery support tools available in the app (the app must send push notifications to get credit for this item, even if a general push notification to use the app)?
4. Does the app check in with the app user regarding their recovery (e.g., questions to assess how you feel)?
5. Does the app track and calculate recovery related information (e.g., counting days of not using, keeping track of daily recovery tasks, money saved)?
6. Does the app function as a journal for recovery-related journaling?

- Tools for tracking and navigating cravings

1. Does the app provide a tracker for cravings (i.e., app users note in the app their triggers for using or for craving; our SiS cig log is a craving tracker, as it generates the pie chart of triggers)?
2. Does the app help users identify places to avoid?
3. Does the app notify users when they are near self-identified areas to avoid (i.e., geo-fencing, proximity warnings, trigger reminders for specific locations such as bars, dispensaries, liquor stores, etc.)?
4. Does the app offer distraction tools (i.e., these tools are specifically offered to provide distraction from cravings)?
5. Does the app nudge app user to engage in substance-free enjoyable activities, to stay in recovery?
6. Does the app assign/provide mindfulness exercises (including prayer)?
7. Does the app connect the app user with emergency contacts (e.g., best friend, call to clinician, AA sponsor, care team; the app needs to ask the app user to specify this contact in order for this to be marked YES)?
8. Does the app provide any other positive psychology tools (e.g., remember good things, discover strengths, musical breaks, laughter exercises, observe kindness, or other)?
9. Does the app provide a gratitude journaling function?

- Limited access

1. Is payment necessary for any content?
2. Is the app limited to private invite (e.g., only if you are a patient at that clinic, part of a community)?
